# Management of paediatric illnesses by patent and proprietary medicine vendors in Nigeria

**DOI:** 10.1186/s12936-015-0747-7

**Published:** 2015-06-04

**Authors:** Emily Treleaven, Jenny Liu, Lisa M. Prach, Chinwoke Isiguzo

**Affiliations:** Global Health Sciences, University of California, San Francisco, 550 16th Street, Mission Hall, Global Health & Clinical Sciences Building, San Francisco, CA 94158 USA; Department of Social and Behavioral Sciences, University of California, San Francisco, 3333 California St., Suite 455, San Francisco, CA 94143 USA; Society for Family Health, No. 8 Port Harcourt Crescent, Area 11, Garki, Abuja Nigeria

**Keywords:** Malaria, Pneumonia, Diarrhoea, Nigeria, Private sector, Drug vendors, Integrated management of childhood illnesses, Community case management

## Abstract

**Background:**

In Nigeria and elsewhere, informal drug sellers, or patent and proprietary medicine vendors (PPMVs), are a common source of care for children with malaria, diarrhoea, and pneumonia. However, their knowledge and stocking of recommended treatments for these common childhood illnesses are not well understood.

**Methods:**

A census of PPMV shops was conducted in Kogi and Kwara states. A shop survey was conducted on a subset of 250 shops. Multivariate regression analysis was used to assess associations between shop worker characteristics and (1) knowledge of optimal treatments for malaria, diarrhoea, and pneumonia, and (2) stocking of essential medicines to treat these illnesses.

**Results:**

From the census, 89.9 % of shops stocked oral rehydration solution (ORS), while 61.1 % of shops stocked artemisinin-based combination therapies and 72.2 % of shops stocked amoxicillin. Stocking patterns varied by state, urban/rural location, and according to whether or not the shop was headed by someone with formal health training (e.g. having a professional health education degree). In multivariate analyses, selling drugs wholesale and participating in any training in the past year was associated with a higher likelihood of naming the correct treatment for malaria, and having formal health training was associated with stocking ORS. However, few other PPMV characteristics were predictive of correct knowledge of optimal treatments and stocking behaviour.

**Conclusion:**

Many PPMVs lack the knowledge and tools to properly treat common childhood illnesses. PPMV knowledge and selling of essential medicines for these illnesses should be strengthened to improve child health in Nigeria.

## Background

Pneumonia, diarrhoea, and malaria are the leading causes of child mortality in Nigeria, from which over one million children under five are estimated to die each year [1]. These conditions contribute 16.4, 18.7, and 20.1 % of the total burden of disease, respectively [1]; thus, ensuring prompt and proper diagnosis and treatment for these illnesses is essential for reducing childhood morbidity and mortality [2].

Treatment guidelines in Nigeria and elsewhere recommend that children exhibiting signs of pneumonia, including coughing, fever, and rapid breathing, be seen by a health professional, and if properly diagnosed, prescribed amoxicillin in dispersible tablet form [3]. Children diagnosed with uncomplicated malaria should be treated with artemisinin-based combination therapy (ACT) after, ideally, parasitological confirmation [4, 5]. Children with diarrhoea should be given low-osmolarity oral rehydration solution (ORS) in combination with zinc tablets to reduce the duration and severity of fluid loss [6].

The Integrated Community Case Management (iCCM) strategy for childhood illnesses adopted by the Nigeria Ministry of Health (MOH) in 2014 aims to improve access to these treatments and provide referrals to formal care when necessary through community-based health workers or other resource persons [7]. The MOH, in the early stages of implementing its iCCM strategy, defines iCCM resource persons as respected community members, aged 18 to 65, and literate, and who are accessible and willing to participate [7]. Providers eligible to deliver iCCM include for-profit drug retailers, or patent and proprietary medicine vendors (PPMVs), who are often the first source of care for common health concerns and commodities [8, 9], including treatment for children under five with fever or cough [10]. With an estimated over 200,000 shops in Nigeria [11], PPMVs are highly accessible and are a particularly important access point for basic health services among rural and poor households [8, 12, 13].

However, PPMV licensure does not require formal pharmacy training or a minimum level of education [14–16], which raises concerns about the quality of care that PPMVs provide. PPMV licensure is currently under review by the Pharmacy Council of Nigeria (PCN), and some PPMVs have formal medical training, such as nursing or pharmacy. PPMVs are permitted to sell pre-packaged, over-the-counter drugs (e.g. ACT, ORS) but not prescription drugs (e.g. antibiotics) [17]. Past studies of PPMVs in Nigeria have found they may improperly identify and treat common childhood illnesses [18, 19], and that many PPMVs sell drugs and services beyond their legal scope of practice [11, 16]. Because PPMVs are for-profit microenterprises, they are particularly responsive to what their customers ask of them [14, 20], which may lead to under-stocking and under-provision of low-demand products, such as zinc [21]. Despite their central role in delivering treatments for childhood illnesses [10]. Little is known about how PPMVs provide integrated disease management for children [22]. The few existing studies of PPMV practices around childhood illness focus almost exclusively on malaria [16, 23, 24], with only one study on childhood diarrhoea [25] and none on pneumonia [22].

Because of their abundance, accessibility, and eligibility as an iCCM provider, coupled with concerns over quality of care, this study of PPMV practices examines their stocking practices and knowledge for three common childhood illnesses, malaria, diarrhoea, and pneumonia, for which there is scant existing evidence. Specifically, this paper aims to describe PPMVs’ stocking patterns of essential medicines to treat malaria, pneumonia, and diarrhoea, and assess associations between shop worker characteristics and knowledge and stocking of optimal treatments for these diseases. Understanding PPMVs’ knowledge and stocking of malaria, pneumonia, and diarrhoea treatments in the context of iCCM scale-up in Nigeria is important as they are a primary point of care for sick children; implications for iCCM implementation are discussed.

## Methods

### Data sources

A census of all retail establishments selling drugs in Kogi and Kwara states was conducted from April to September 2013. Kogi and Kwara states were chosen to represent Nigeria’s central region, with mixed populations by religion, socio-economic status, ethnicity, and mix of urban, peri-urban, and rural areas, in comparison to southern Nigeria, where a majority of studies in Nigeria are concentrated [24].

This census listing produced a database of 2,083 shops (1,088 in Kogi, 995 in Kwara), and collected limited information on shop owner and worker characteristics, and the types, doses, and brands of medicines stocked for malaria, pneumonia, and diarrhoea. From this listing, 250 shops (125 in each state) were selected for a survey that collected additional information on shop characteristics, services, and knowledge and practices of shop workers. The sub-sample included in the survey was not meant to be representative of the census, but to generate an analytical sample. Due to logistical challenges and security concerns of extended periods of stay in certain areas, the sub-sample was chosen through a modified stratified random sampling approach. In each state, shops within a 25-km radius of the center of four urban or peri-urban hubs (ranging from 21 to 39 shops per hub) were randomly selected, stratified across urban, peri-urban, and rural locations (Kogi: Ayangba, Ida, Lokoja, and Okene; Kwara: Ajase-Ipo, Bode-Sadu, Ilorin, and Omu-Aran). In consultation with a geospatial expert, a 25-km was determined to be sufficient to generate a difference in urban, peri-urban, and rural locations. If a shop could not be located (due to absent provider, closed shop, or incorrect address), the next closest shop was selected as a substitute (Kogi: 30 shops, Kwara: 24 shops) to preserve sample stratification, regardless of its inclusion on the census, and to account for shops that opened after the census was taken. All shops selected in the sampling process or identified as a substitute were eligible to participate.

### Data collection

From Monday to Saturday in September and October 2013, one multi-lingual study surveyor was assigned to visit one shop per day. Upon arrival, the surveyor self-identified as working with a non-profit organization focused on health, and sought consent from the person in charge (79.2 % shop owner, 6.8 % manager, 13.2 % apprentice, 0.8 % other). Shop owners were not given advance notice. All shops located and approached for consent agreed to participate.

Surveyors administered the survey in the morning when customer traffic was typically low. Interruptions for attending to customers were permitted. The survey instrument was designed to collect information on shop owners’ and workers’ sociodemographic characteristics, self-reported knowledge and practices for treating childhood malaria, diarrhoea, and pneumonia, resources for drug and healthcare advice, and training. Participants were given a small gift for participation (wall clock valued at 500 Naira [~US$3.13]).

### Outcome measures

Two outcome measures were analyzed in this paper: (1) correct knowledge of the most effective drug to treat malaria and diarrhoea; and (2) whether the shop stocked at least one effective drug for the treatment of malaria and diarrhoea. Due to the extremely low recognition and understanding of pneunomia among respondents, multivariate analyses of outcomes for pneumonia were not possible (see results). Further, diagnosis is excluded as an outcome because PPMVs are not legally allowed to perform diagnostic tests. Knowledge of the most effective drug to treat childhood malaria, diarrhoea, or pneumonia was coded as correct if the respondent answered with ACT, ORS, or amoxicillin, respectively. While other appropriate treatments exist for these illnesses, ACT, ORS, and amoxicillin were chosen because they are listed as optimal treatments under iCCM guidelines. Stocking of zinc for treatment of diarrhea is excluded as an outcome due to its extremely low prevalence in the study area. Respondents were asked about treatments for specific diseases and symptoms; where they provided brand names, responses were recoded to the type of medicine, such as “ACT” for “Coartem.”

### Data analysis

In bivariate analysis, differences in knowledge of most effective treatments, drug stocking, shop characteristics [state, location (urban, peri-urban, or rural), registration with the PCN, selling wholesale drugs, and offering tests or examinations to customers], and respondents’ characteristics (age, sex, education, years of experience working at a drug shop, religion, proportion of household income from the shop, and participation in any type of training in the past year) between respondents with and without formal health training are examined. Respondents self-reporting health training as a doctor, nurse, midwife, pharmacist, laboratory technician, or community health extension worker (CHEW) were considered to have formal health training, while current or finished PPMV apprentices were considered not to have formal health training. In addition to retail sales, some PPMVs sell drugs wholesale to other PPMVs or pharmacies. Respondents self-reported whether they provide examinations or diagnostic tests, such as malaria rapid diagnostic tests or physical examinations. Respondents who reported that more than half of their household income was generated from the PPMV shop were coded as having the majority of their household income come from the shop.

For treatment knowledge and stocking outcomes, multivariate analyses were conducted using logistic regressions. It is important to note that too few respondents gave correct answers for pneumonia treatment, which precluded further multivariate analysis (see results). Covariates for PPMV shop and worker characteristics were selected based on a priori hypotheses of determinants of knowledge and stocking practices and included those listed above. Analyses were conducted using STATA SE Version 13.1.

Interaction effects were tested to explore whether PPMVs with formal health training had significantly different knowledge or stocking practices if they were male, had more years of experience, or were located in an urban area. As none were significant, they are not included in the final analysis. Alternative categorizations of age, years of experience, and profession were examined to assess sensitivity, but resulting estimates did not change substantive findings. It is important to note that the Affordable Medicines Facility-malaria (AMFm) programme, an international subsidy on ACTs to increase its supply and affordability and reduce the use of non-recommended anti-malarials, which included a malaria and malaria training programme for participating providers, operated in the study area [20]. Selected retail outlets, including some larger PPMVs, participated in the AMFm programme as distributors, and many PPMVs were recipients of the subsidized retail products and training component. We explored the independent effect of AMFm inclusion, but, because participation in the AMFm program was not associated with our outcome measures, it is not included in the final analysis.

### Ethical considerations

Ethical approval was obtained from the National Health Research Ethics Committee of Nigeria (NHREC/01/01/2007-05/09/2013). Surveyors obtained written informed consent from all persons in charge at selected shops.

## Results

### Stocking patterns from the PPMV shop census

**Fig. 1:**
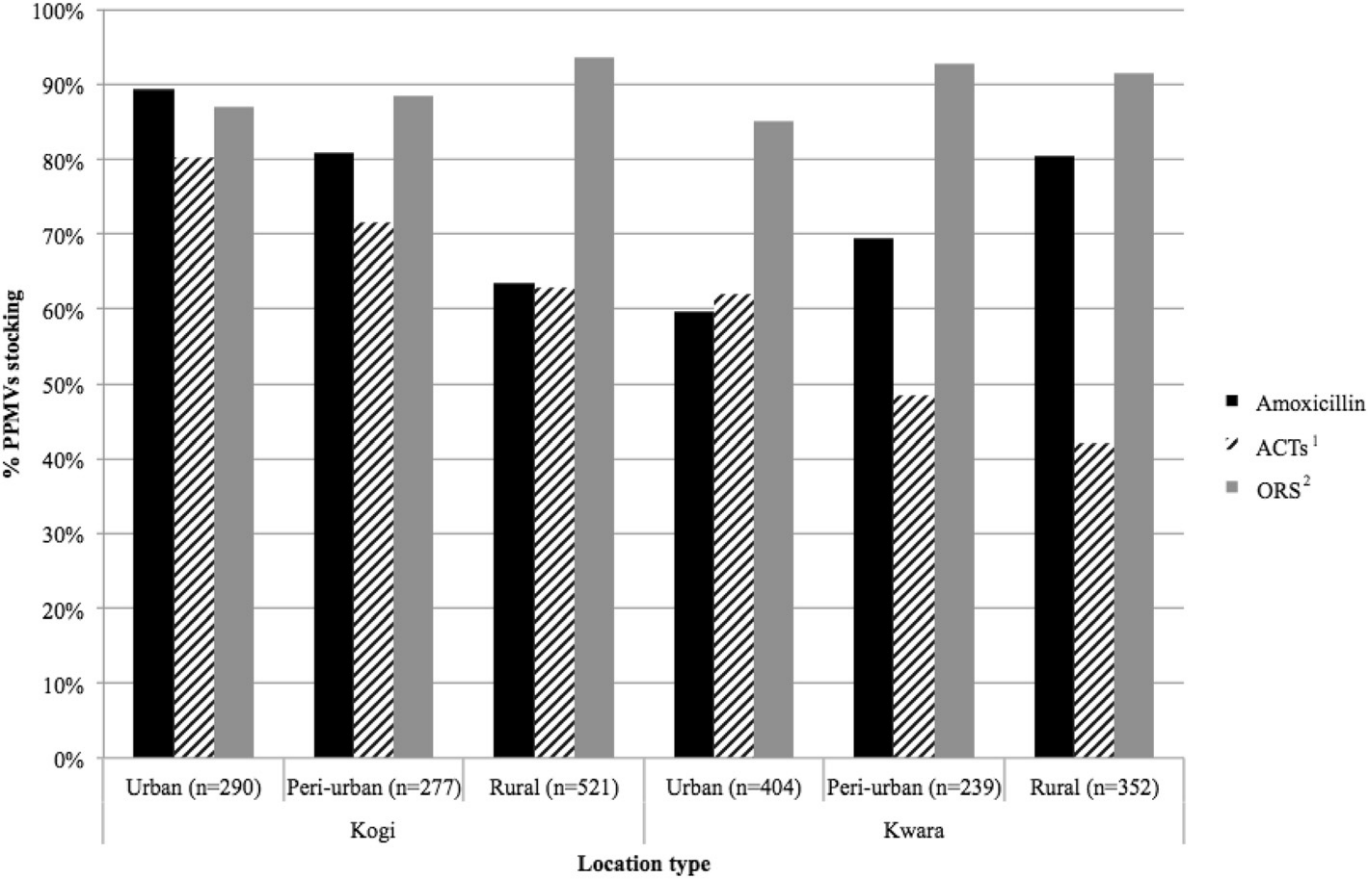
Stocking of essential medicines by location at Kogi and Kwara PPMVs. The percentage of PPMVs (*n* = 2083) stocking different essential medicines for the treatment of childhood illnesses was plotted according to state and location type within state. Within each drug or commodity type, all brands and dosages are grouped together (Source: Census). ^1^ACTs = artemisinin-based combination therapy ^2^ORS = oral rehydration solution

A total of 1,088 PPMV shops in Kogi and 995 in Kwara were identified during the census. 33.3 % of Kogi shops and 12.2 % of Kwara shops participated in the AMFm programme. Figure 1 displays the stocking patterns for essential medicines across urban, peri-urban, and rural locations. In Kogi, stocking of any form of amoxicillin was higher in urban areas (89.3 %) compared to peri-urban (80.9 %) and rural (63.5 %) areas. In Kwara, the trend for amoxicillin stocking was reversed: 59.7 % among urban PPMVs, increasing to 69.5 % of peri-urban shops and 80.4 % of rural shops. Although lower in Kwara, stocking of ACTs in Kogi and Kwara was highest in urban areas (80.3 and 61.9 %, respectively) compared to peri-urban (71.5 and 48.5 %, respectively) and rural (62.8 and 42.1 %, respectively) shops. Of the essential medicines, availability of ORS was highest overall, with 85.0 % (urban Kwara) to 93.5 % (rural Kogi) of PPMV shops stocking it. Less than 3 % of all PPMVs stocked zinc tablets. Zinc stocking was highest in urban Kogi, 8.8 %, but fewer than 1 % of PPMVs stocked zinc in urban Kwara.

**Fig. 2:**
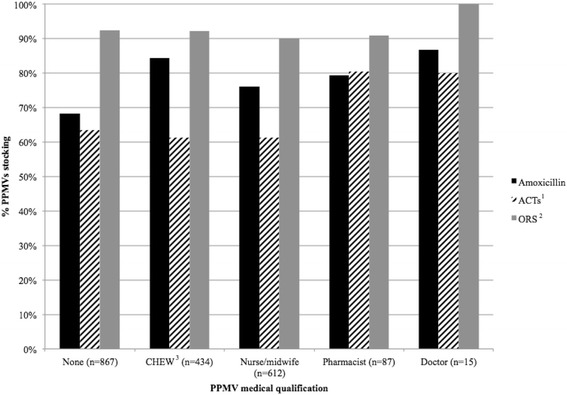
Stocking of essential medicines by formal medical training at Kogi and Kwara PPMVs. The percentage of PPMVs (*n* = 2015) stocking different essential medicines for the treatment of childhood illnesses was plotted according to the shop owner’s self-reported type of formal health training. Within each drug or commodity type, all brands and dosages are grouped together (Source: Census). ^1^ACTs = artemisinin-based combination therapy ^2^ORS = oral rehydration solution ^3^CHEW = community health extension worker

Overall, 57.0 % of PPMVs had some type of formal health training, of whom 30.4 % were nurses or midwives, 21.5 % CHEWs, 4.3 % pharmacists, and 0.7 % doctors. Among shop owners and workers with formal health training, over three-quarters (86.7 % of doctors, 75.9 % of nurses/midwives, 84.3 % of CHEWs, and 79.3 % of pharmacists) stocked amoxicillin, while only 68.2 % of PPMVs without formal health training did so (Fig. 2). A higher percentage of shop owners who were pharmacists or doctors stocked ACT (80.4 % and 80.0 %, respectively), compared to PPMVs who were CHEWs (61.3 %), nurses/midwives (61.3 %), and those without formal health training (63.3 %). Stocking of ORS was very high overall, with 100 % of doctors and over 90 % of all other shop owner types stocking it. Stocking of zinc was highest among nurses/midwives (12.7 %) and pharmacists (8.5 %), but was less than 3 % of CHEWs and those without formal health training, and no doctors stocked zinc.

### Shop survey sample characteristics

For the subset of PPMVs shops selected for the shop survey, basic characteristics of the shop and the person in charge are summarized in Table 1. Overall, 21.2 % of PPMVs in the shop survey had any formal health training. The percentage of PPMVs with formal health training differs between the census and the shop survey due to two reasons: (1) The random sampling frame employed in the shop survey had to be modified to accommodate logistical challenges and security risks; (2) Some shops enumerated from the census were not able to be located if they were no longer in business, and replacement shops may include new businesses that were newly operational since the census was taken. Limitations are discussed in the following section.

**Table 1:** PPMV Shop characteristics (Source: Shop survey)^a^

	Health training n (%)	No health training (%)	*p*-value
Knowledge of shop workers and owners			
Named most effective treatment for malaria	39 (73.6)	135 (68.5)	
Named most effective treatment for diarrhoea	15 (32.0)	63 (28.3)	
Named most effective treatment for pneumonia	0 (0.0 %)	1 (0.5 %)	
Drug stocking^b^			
Stocked ACT^c^ for malaria treatment	38 (71.7)	144 (73.9)	
Stocked ORS^d^ for diarrhoea treatment	45 (84.9)	146 (74.9)	
State			
Kogi	21 (39.6)	103 (52.3)	
Kwara	32 (60.4)	94 (47.7)	
Location			
Urban	11 (20.8)	59 (30.0)	
Peri-urban	17 (32.1)	68 (34.5)	
Rural	25 (47.2)	70 (35.5)	
Male respondents^e^	28 (52.8)	117 (60.3)	
Age of respondent^b^ mean ± SD (years)	40.0 ± 1.7	31.0 ± 7.5	0.000
Religion^a^			
Christian	36 (67.9)	115 (59.0)	
Muslim	17 (32.1)	80 (41.0)	
Education			0.000
Primary	1 (1.9)	12 (6.1)	
Secondary	2 (3.8)	139 (70.6)	
Post-secondary	50 (94.3)	46 (23.4)	
Profession/Formal health training^b^			
Current PPMV apprentice or no training	-	57 (32.3)	
Finished PPMV apprentice	-	132 (67.7)	
CHEW^f^	28 (52.8)	-	
Laboratory technician	4 (7.6)	-	
Pharmacist	3 (5.7)	-	
Nurse or midwife	18 (34.0)	-	
Mean years of experience working at a PPMV shop^b^	16.7 ± 1.6	10.5 ± 0.6	0.000
Proportion of household income from PPMV shop			
Half or less	30 (56.6)	89 (45.2)	
More than half	23 (43.4)	108 (54.8)	
Shop sells wholesale drugs^g^	8 (15.1)	37 (18.9)	
Member of PCN	11 (20.8)	35 (17.8)	
Shop offers tests or examinations to customers	28 (52.8 %)	59 (30.3 %)	0.002
Participated in any trainings in past 12 months	36 (69.2 %)	124 (66.3 %)	
Number of observations	53	197	

^a^Patent and Proprietary Medicine Vendors

^b^ *n* = 248

^C^artemisinin-based combination therapy

^d^oral rehydration solution

^e^ *n* = 247

^f^community health extension worker

^g^ *n* = 249

A majority of formally trained PPMVs were CHEWs (52.8 %), followed by nurses/midwives (34.0 %), laboratory technicians (7.6 %), and pharmacists (5.7 %). Among PPMVs without health training, 67.7 % had completed an apprenticeship; 32.3 % were current PPMV apprentices or did not report any form of formal or informal training. In this sample, a higher percentage of PPMVs in Kwara had health training (60.4 % versus 39.6 % in Kogi), while PPMVs without formal health training were evenly distributed between the two states. A slightly higher proportion of health-trained PPMVs were in a rural area (47.2 % versus 20.8 % and 32.1 % in urban and peri-urban areas, respectively). Those without formal training were more evenly distributed across locations.

Over half of health-trained and untrained PPMVs were male (52.8 and 60.3 %, respectively). The mean (SD) age of health-trained respondents was nine years older than untrained respondents—40.0 years (12.3) compared to 31.0 years (10.9), respectively (*p* = 0.000). A higher percentage of PPMVs were Christian than Muslim. Both educational attainment and experience working at a PPMV shop were significantly higher among respondents with health training than those without: 94.3 % versus 23.4 % with post-secondary education (*p* = 0.000), respectively; a mean (SD) of 16.7 years (11.2) of PPMV work experience versus 10.5 years (8.2; *p* = 0.000), respectively. Health-trained PPMVs were significantly more likely to offer tests or examinations to customers (52.8 %) than untrained PPMVs (30.3 %; *p* = 0.002). Differences between health-trained and untrained PPMV were not observed for respondents who reported that more than half of their household income came from the shop, that their shop sold drugs wholesale, or that the shop was registered with the PCN.

Differences between respondents with and without formal health training were not statistically significant for naming of the most effective treatments for malaria, diarrhoea, or pneumonia, or stocking of ACT, ORS, or amoxicillin.

### Multivariate analyses of the correlates of knowledge and stocking of most effective treatments

In multivariate analysis (Table 2), characteristics associated with having higher odds of correctly naming the most effective treatment for malaria included residing in Kogi state (OR = 4.093; 95 % CI 1.711–9.793), being a PCN member (OR = 3.181;95 % CI 1.165–8.685), selling drugs wholesale (OR = 4.230; 95 % CI 1.351– 13.680), and having participated in any trainings in the previous year (OR = 2.397; 95 % CI 1.135 - 5.060). No PPMV characteristics were significantly associated with stocking ACTs. State was the only characteristic associated with naming the most effective treatment for diarrhoea (Table 3): respondents in Kogi were significantly less likely to identify ORS than those in Kwara (OR = 0.240; 95 % CI 0.104 - 0.555). Respondents with formal health training were significantly more likely to stock ORS than those without health training (OR = 2.625; 95 % CI 1.103 - 6.802). No other covariates significantly predicted stocking of ORS. Paediatric formulations of amoxicillin are not available in Nigeria; therefore, any reported amoxicillin stocking, which is against current PPMV regulations, is for adult formulations. For this reason, further analysis of this outcomes was excluded.

**Table 2:** Multivariable logistic regression of the likelihood of the PPMV’s knowledge and stocking of most effective treatments for malaria. (Source: Shop survey)

	Named most effective malaria treatment (*n* =229)		Stocked ACT (*n* =229)	
	Odds Ratio (95 % CI)	*p*-value	Odds Ratio (95 % CI)	*p*-value
Kogi (*vs* Kwara)	4.093*** (1.711–9.793)	0.002	0.702 (0.309 - 1.597)	0.399
Urban location (*vs* rural)	1.794 (0.782–4.093)	0.165	0.713 (0.331 - 1.537)	0.388
Peri-urban location (*vs* rural)	1.073 (0.486 - 2.370)	0.862	1.346 (0.610 - 2.968)	0.462
Age (years)	0.975 (0.933 - 1.020)	0.273	0.969 (0.930 - 1.012)	0.152
Has formal health training (*vs* none)	1.658 (0.694 - 3.959)	0.255	0.650 (0.286 – 1.477)	0.304
Years of experience	1.038 (0.979 - 1.101)	0.207	1.048 (0.991 - 1.108)	0.104
Muslim respondent (*vs* Christian)	0.564 (0.283–1.122)	0.103	0.670 (0.332 - 1.354)	0.265
PCN member (*vs* not a member)	3.181** (1.165–8.685)	0.024	1.190 (0.524 - 2.703)	0.677
Majority of household income is from shop (*vs* no)	1.047 (0.521 - 2.103)	0.897	0.540 (0.264 - 1.105)	0.149
Shop sells wholesale drugs (*vs* no)	4.230** (1.351– 13.680)	0.014	0.643 (0.282 - 1.466)	0.293
Participated in any trainings in previous year (*vs* no)	2.397** (1.135 - 5.060)	0.022	1.444 (0.723 - 2.873)	0.295
Shop offers tests or examinations to customers (*vs* no)	0.763 (0.383 - 1.520)	0.442	1.578 (0.796 – 3.128)	0.192

* *p* < 0.1, ** *p* < 0.05, *** *p* < 0.01

**Table 3:** Multivariable logistic regression of the likelihood of the PPMV’s knowledge and stocking of most effective treatments for diarrhoea. (Source: Shop survey)

	Named most effective diarrhoea treatment (*n* =229)		Stocked ORS (*n* =229)	
	Odds ratio (95 % CI)	*p*-value	Odds ratio (95 % CI)	*p*-value
Kogi (*vs* Kwara)	0.240*** (0.104 - 0.555)	0.001	1.524 (0.655 - 3.544)	0.328
Urban location (*vs* rural)	0.844 (0.376 - 1.893)	0.681	1.237 (0.549 - 2.791)	0.608
Peri-urban location (*vs* rural)	1.272 (0.598 - 2.705)	0.532	1.093 (0.493 - 2.424)	0.827
Male (*vs* female)	0.563 (0.258 - 1.228)	0.149	0.783 (0.346 - 1.771)	0.556
Age (years)	1.004 (0.961 - 1.049)	0.862	0.966 (0.925 - 1.009)	0.118
Has formal health training (*vs* no)	0.551 (0.237 - 1.279)	0.165	2.625** (1.013 - 6.802)	0.047
Years of experience	0.996 (0.942 - 1.053)	0.888	1.017 (0.960 - 1.078)	0.565
Muslim respondent (*vs* Christian)	0.836 (0.420 - 1.664)	0.609	0.613 (0.305 - 1.229)	0.168
PCN member (*vs* no)	1.690 (0.774 - 3.692)	0.188	1.849 (0.731 - 4.682)	0.194
Majority of household income is from shop (*vs* no)	1.143 (0.573 - 2.279)	0.704	1.322 (0.655 - 2.670)	0.436
Shop sells wholesale drugs (*vs* no)	0.565 (0.227 - 1.409)	0.221	1.923 (0.706 - 5.233)	0.201
Participated in any trainings in previous year (*vs* no)	1.538 (0.746 - 3.250)	0.249	1.253 (0.608 - 2.582)	0.540
Shop offers tests or examinations to customers (*vs* no)	1.119 (0.576 – 2.171)	0.739	1.076 (0.537 - 2.157)	0.836

* *p* < 0.1, ** *p* < 0.05, *** *p* < 0.01

## Discussion

Drug retailers, such as PPMVs, provide a majority of drugs to treat childhood illnesses in low- and middle-income countries and have the potential to contribute to advancing child health. However, this and other studies [12, 19, 26] have indicated that PPMVs do not always properly treat common childhood illnesses. In Kogi and Kwara states, about 75 % of shops stocked ORS and ACT, the majority of shops offered amoxicillin contrary to current regulations, and those stocking zinc were minimal.

The results also indicate that although many PPMVs stock treatments for pneumonia, diarrhoea, and malaria, knowledge of the most effective treatment is lower. Moreover, having formal health training was not significantly associated with naming the most effective treatments for malaria or diarrhoea in multivariate analyses, and was only statistically related to stocking of ORS. Hence, even though some PPMVs have formal health training, it does not consistently relate to increased knowledge of optimal treatment or stocking behaviour. For PPMVs to be engaged in delivering iCCM, further training is needed to increase basic health knowledge and improve stocking and selling practices. Further research should be undertaken to identify optimal training methods to increase PPMV knowledge.

These findings corroborate other reports that PPMVs sell a range of medicines beyond their scope of service, including antibiotics [11, 27, 28], yet under-stock low-demand products [23]. Concerted campaigns to increase awareness of prompt treatment for malaria in combination with supply-side drug subsidies have bolstered the sales of ACTs and resulted in a higher proportion of shops stocking ACTs across Nigeria [20, 29]. Broader customer demand and awareness of the correct treatments for other paediatric illnesses are needed to reinforce optimal PPMV stocking and selling behaviours. For example, caregiver knowledge of symptoms and treatment for diarrhoea remains low [23, 30], and a similar campaign to generate demand for proper care-seeking and treatment from qualified and trained providers is needed in concert with increased zinc supply at PPMVs.

Current efforts to implement iCCM in Nigeria must consider these factors in advance of programme scale-up. In particular, according to the current implementation framework, PPMVs are eligible to become community-based resource persons trained and certified to deliver iCCM. Elsewhere, trained community health workers have successfully participated in iCCM efforts. In Ghana and Uganda, lay community health workers trained in dosing and referral for treatment of fever and pneumonia, respectively, provided appropriate treatment for a majority of cases among children under five years of age [31, 32]. In Uganda, drug retailers trained in iCCM and given diagnostic supplies and pre-packaged child formulations of ACT, amoxicillin, and ORS and zinc correctly managed childhood illnesses in 87.7 % of cases [33]. This suggests that PPMVs may serve as an effective lay cadre of iCCM providers in Nigeria, if properly trained. However, current PPMV regulations are inconsistent with iCCM guidelines, which allow certified providers to treat uncomplicated diarrhoea, malaria, and pneumonia with ORS and zinc, ACT, and amoxicillin, respectively. The prohibition of antibiotic sales at PPMVs precludes legally training them on the proper treatment of pneumonia or other bacterial infections, as well as enforcing regulatory mechanisms to ensure medicines are of high quality and properly formulated for children. While the large existing numbers of PPMVs in Nigeria could be leveraged to quickly increase access to these basic paediatric services, regulations must be harmonized with national policies and iCCM guidelines to provide PPMVs with the training necessary to effectively treat uncomplicated episodes of the most common paediatric illnesses, and recognize and refer when necessary.

Several limitations of this study should be noted. The sample of PPMVs was limited by security and logistical concerns and was not representative of the census, which may reduce the external generalizability of the paper’s results. While the census indicated over half of PPMVs had formal health training, only 20 % in the shop survey sample reported this. However, the trends in formal health training are consistent with the census in terms of state and location type as part of the sampling stratification. Training and other sociodemograhic characteristics are self-reported. Questionnaires were written in English and administered in several local languages, allowing for potential errors in translation. Stocking practices of essential medicines did not record which formulations were sold or whether the formulation stocked was an approved form. Despite these challenges, this paper provides the most comprehensive assessment of knowledge and stocking for the integrated treatment of childhood malaria, diarrhoea, and pneumonia at PPMVs in Nigeria to date.

## Conclusions

PPMVs may provide an opportunity to improve children’s health at the community level, but several challenges remain, including sub-optimal stocking and selling practices. Although PPMVs are included as implementers of iCCM under current guidelines, their role as community resource persons remains unclear. There is a need to ensure PPMVs understand how to correctly administer the essential medicines they stock through improved training mechanisms, in order to effectively increase access to quality-assured paediatric care in Nigeria.

## Abbreviations

ACT: Artemisinin-based combination therapy
CHEW: Community health extension worker
iCCM: Integrated community case management
MOH: Ministry of health
NPC: National population commission
ORS: Oral rehydration solution
PCN: Pharmacy council of nigeria
PPMV: Patent and proprietary medicine vendor
UNICEF: United Nations children’s fund
WHO: World Health Organization
